# Trajectories of Unhealthy Behaviors in Midlife and Risk of Disability at Older Ages in the Whitehall II Cohort Study

**DOI:** 10.1093/gerona/glw060

**Published:** 2016-03-30

**Authors:** Fanny Artaud, Séverine Sabia, Aline Dugravot, Mika Kivimaki, Archana Singh-Manoux, Alexis Elbaz

**Affiliations:** ^1^INSERM, U1018, Centre for Research in Epidemiology and Population Health, Villejuif, France.; ^2^University Versailles St-Quentin, UMRS-1018, Versailles, France.; ^3^Department of Epidemiology and Public Health, University College London, UK.; ^4^University Paris-Sud, UMRS-1018, Villejuif, France.

**Keywords:** Aging, Cohort study, Disability, Epidemiology, Health behaviors

## Abstract

**Background:**

Most of the evidence on the association between unhealthy behaviors and disability comes from studies in the elderly, where reverse causation and selection bias may distort associations; thus, studies based on midlife trajectories of health behaviors are needed. We examined the association of trajectories of four health behaviors (physical activity, fruit and vegetable consumption, smoking, alcohol), starting in midlife and over 20 years, with subsequent disability risk in early old age (range = 54–84 years) in the Whitehall II cohort study.

**Methods:**

Disability was assessed three times over 3 years. A hierarchical disability indicator was constructed; participants were considered disabled if they reported difficulties with mobility and instrumental activities of daily living or with mobility and instrumental and basic activities of daily living. Behavior trajectories were defined using group-based trajectory models. Multivariable generalized estimating equations logistic models were used to examine their independent associations with disability.

**Results:**

Of 6,825 participants, 19.2% reported being disabled at least once. In mutually adjusted models, participants with persistent inactivity or declining physical activity, recent ex- or current smokers, and persistent/recent abstainers or persistent heavy drinkers had a higher disability risk, whereas fruit and vegetable consumption was not associated with disability. Disability risk increased progressively with the number of unhealthy behavior trajectories: the odds ratio of disability for 2–3 unhealthy trajectories was 2.69 (95% confidence interval = 2.26–3.19); these associations remained after adjustment for a wide range of covariates.

**Conclusions:**

Unhealthy behavior trajectories in midlife are associated with greater disability risk later in life.

Remaining disability-free at older ages is a major challenge in aging societies where the absolute number of disabled persons is expected to increase dramatically due to population aging, despite recent decline in age-standardized prevalence in European countries (1,2). Disability is a major cause of reduced quality of life, hospitalization, institutionalization, and death (3,4). The identification of potentially modifiable risk factors of disability may help develop preventive strategies and slow its progression (5,6).

Previous research suggests that unhealthy behaviors at older ages (physical inactivity, poor diet, smoking, alcohol abstinence, or consumption beyond recommended limits) have an adverse effect on disability (7,8). Much of this evidence comes from studies in the elderly, where reverse causation (disability affecting health behaviors rather than vice versa) and selection bias (sickest subjects are most likely to drop from the study or not to be included in it) may distort associations. Disability at older ages develops progressively over many years, making it important to evaluate putative risk factors in midlife, before the cascade of events leading to disability starts. Studies with a long follow-up are therefore needed to identify trajectories of health behaviors, rather than one-off measures, associated with disability in order to inform preventive strategies (9,10). Accordingly, our aims were to describe trajectories of four midlife health behaviors (physical activity, fruit and vegetable consumption, smoking, alcohol consumption) over 20 years of follow-up, and their association with disability at older ages, using data from the Whitehall II cohort study.

## Methods

### Study Population

The Whitehall II cohort study, established in 1985–1988, is a longitudinal study of 10,308 British civil servants (11). All civil servants aged 35–55 years in 20 London-based departments were invited to participate (participation rate, 73%). The baseline examination (wave 1 [1985–1988]) included a clinical examination and self-administered questionnaire. Subsequent phases alternated between postal questionnaires alone (waves 2 [1988–1990], 4 [1995–1996], 6 [2001], and 8 [2006]) or accompanied by a clinical examination (waves 3 [1991–1993], 5 [1997–1999], 7 [2002–2004], 9 [2007–2009] and 11 [2012–13]). Participants gave informed written consent. Research ethics approvals (University College London [UCL] ethics committee) were renewed at each contact; latest approved was by the Joint UCL/UCLH Committee on the Ethics of Human Research (Committee Alpha, 85/0938).

### Disability

Three disability domains were assessed by questionnaire three times over 8 years (2006–2013): mobility, instrumental activities of daily living (IADL), basic activities of daily living (ADL). Mobility was assessed based on the ability to walk 1/2 mile and climb several flights of stairs. IADLs were assessed based on the ability to prepare hot meals, shop for groceries, make telephone calls, take medications, do work around the house/garden, and manage money. ADLs were assessed based on the ability to bath, dress, eat, get in/out of bed, and use the toilet independently. For each domain, we considered participants as disabled if they were not able to perform ≥1 activities without help. We constructed a hierarchical disability indicator (12) that defines four levels of increasing disability by summing responses to the three dichotomized disability items in a hierarchy (0 = fully independent; 1 = dependent only for mobility; 2 = dependent for mobility and IADLs but not ADLs; 3 = dependent in all domains). Participants not fitting to the hierarchy (4.5%) were excluded. This approach takes three disability domains into account simultaneously and respects the natural history of disability. Few people were disabled in all domains, leading us to compare participants with moderate/severe (score = 2/3) to no/light disability (score = 0/1).

### Health Behaviors

Physical activity, fruit and vegetable consumption, smoking, and alcohol consumption were assessed through self-administered questionnaires five times during the first 20 years of the follow-up (1985–2004), prior to the disability assessment.

*Smoking status* was assessed using questions on current/past cigarette smoking; participants were categorized at each wave as current, ex-, or never smokers.

*Alcohol consumption* was assessed using questions on number of alcoholic drinks (measures of spirits, glasses of wine, pints of beer) in the past 7 days, converted to alcohol units (1 unit = 8g). We categorized alcohol consumption as no/occasional (not in the last week), moderate (women, 1–14 units/wk; men, 1–21 units/wk), heavy (women, ≥ 14 units/wk; men, ≥ 21 units/wk) (13).

At the first three assessments, participants were asked about frequency and duration of participation in mildly energetic (eg, weeding, general housework, bicycle repair), moderately energetic (eg, dancing, cycling, leisurely swimming), and vigorous (eg, running, hard swimming, playing squash) *physical activity*. In 1997–1999 and 2002–2004, the questionnaire included 20 items on frequency and duration of participation in different physical activities (eg, walking, cycling, sports) that allowed to compute hours per week of each level. We categorized these data into recommended level (≥2.5h/wk moderately energetic or ≥1h/wk vigorous physical activity (14)), inactivity (<1h/wk moderately energetic and <1h/wk vigorous physical activity), and intermediate (all others) physical activity.

*Fruit and vegetable consumption* was assessed using the question “How often do you eat fresh fruit or vegetables?” Responses were on an 8-point scale. We classified participants as consuming fruit and vegetable at least twice, once, and less than once daily.

### Covariates

As disability was assessed from 2006 onwards, we used sociodemographic covariates from 2006: sex, age, marital status (married/cohabiting, divorced/separated/widowed, single), socioeconomic status (in 2006 or before retirement for those retired) based on a three-level civil service employment grade representing a comprehensive marker of socioeconomic circumstances (administrative, professional/executive, clerical/support) (15).

Additional covariates were considered time-dependent variables by using data between baseline (1985–1988) and the wave concurrent with the disability assessment. Thus, for disability assessed in 2006, we used data between 1985–1988 and 2006; for disability assessed in 2007–2009, we updated the data until then, and so on. For binary covariates, any report since 1985–1988 placed the participant in the exposed category; for continuous covariates, the mean of all measures since 1985–1988 defined exposure level. Body mass index (BMI = weight/height^2^, kg/m^2^) was categorized as ≤24.9 (normal), 25.0–29.9 (overweight), and ≥30kg/m^2^ (obese). Cognition was assessed using the mini-mental state examination, with higher scores corresponding to better function. Depressive symptoms were derived from the 30-item General Health Questionnaire, with scores ≥4 corresponding to high depressive symptoms (16), or antidepressant drugs use. Bone fractures were self-reported. Chronic conditions included diabetes (doctor-diagnosed diabetes or fasting glucose ≥7.0 mmol/L or diabetes medication), anti-inflammatory drugs use for joint pain in the last 14 days, cancer (linkage to the National Health Service’s cancer register), drugs use for osteoarthritis or rheumatic arthritis in the last 14 days, and Parkinson’s disease. Cardiovascular disease and risk factors included stroke and coronary heart disease (17), hypertension (systolic blood pressure ≥ 140mm Hg or diastolic blood pressure ≥ 90mm Hg or antihypertensive medication), and hypercholesterolemia (total cholesterol level ≥ 7.25 mmol/L or lipid lowering drugs).

### Statistical Analysis

Health behavior trajectories over 20 years were defined using group-based trajectory models, using data from all participants with at least one assessment over five waves (10,205–10,301 participants depending on the health behavior; on average, participants had 3.9 measures). This method allows individuals with similar trajectories to be grouped (18). We tested models including two to five trajectories. Two criteria were used to determine the optimal number of trajectories: Bayesian information criterion (lower absolute values correspond to better fit); posterior probabilities of group assignment (the likelihood that an individual belongs to a given trajectory; all trajectories should have a mean posterior probability ≥ 0.70 and each trajectory contain ≥5% of participants). This method was implemented with Proc TRAJ (SAS 9.3) (19).

We excluded participants who could not be assigned to at least one trajectory of health behaviors and those with no data on disability status or covariates. We described participants’ characteristics as a function of disability status (disabled at least once between 2006 and 2013), and number of unhealthy behavior trajectories.

Generalized estimating equations (GEE) logistic models were used to study the association between health behavior trajectories and subsequent disability (moderate/severe vs no/light disability) assessed three times. Models were adjusted for sociodemographic covariates (sex, age in 2006, marital status, socioeconomic status), continuous time in years since 2006, and the time × age interaction. Interactions between time and health behavior trajectories were nonsignificant (*p*-values > .13) and not retained. We first built a separate model for each health behavior (Model 1); based on these analyses, we defined unhealthy and healthy trajectories for each behavior and used these in further analyses. We then included all behaviors in a single multivariable model, either as categorical or binary variables (Model 2), to estimate their mutually adjusted association with subsequent disability. A fully adjusted model (Model 3) further included time-dependent covariates (BMI, mini-mental state examination, depressive symptoms, psychotropic drugs, bone fracture, chronic conditions, cardiovascular disease, and risk factors).

We examined the association between disability and an unhealthy behavior trajectories score that represents the number of unhealthy behavior trajectories (range, 0–3) independently associated with disability (Model 3). As few participants had three unhealthy trajectories (3%), we combined them with those with two.

In sensitivity analyses, we examined each disability domain separately; as few participants were disabled for ADLs, they were combined with those disabled for IADLs (20). Because it has been reported that the association between health behaviors and disability are different in obese and nonobese persons (8), we conducted additional analyses stratified by obesity status in 2006. To take into account the ordinal nature of the disability indicator, we used multinomial GEE models, with the hierarchical score in four classes as the dependent variable. We also took death and dropout into account using an inversely probability-weighted GEE approach (21) and examined the impact of excluding participants with a single health behavior assessment.

Analyses were performed using SAS 9.3–9.4 (SAS Institute Inc, Cary, NC) and R3.0 (R-Foundation for Statistical Computing, Vienna, Austria); *p*-values are two-sided and *p* ≤ .05 considered statistically significant.

## Results

**Figure 1.**
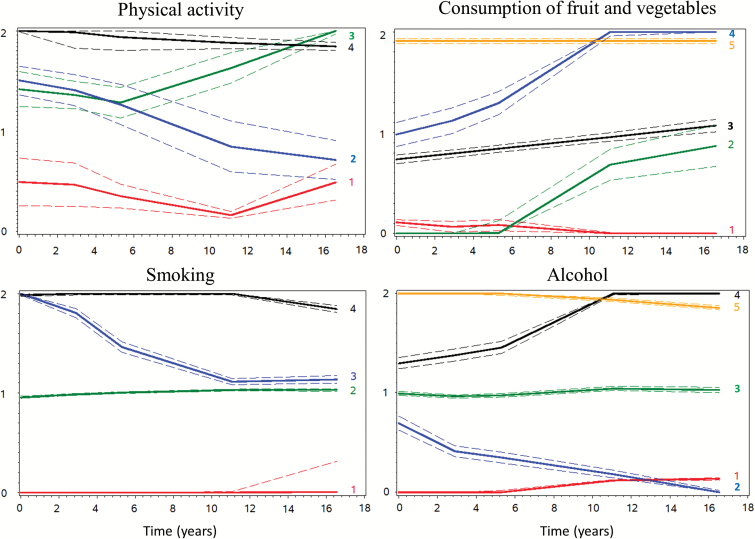
Health behavior trajectories (1985–2004). Trajectories (95% confidence intervals) for physical activity (*n* = 10,205; 0 = inactivity, 1 = intermediate, 2 = recommended), fruit and vegetable (*n* = 10,301; 0 = low, 1 = intermediate, 2 = high), smoking (*n* = 10,295; 0 = never, 1 = ex-smoker, 2 = current smoker), alcohol (*n* = 10,291; 0 = no/occasional, 1 = moderate, 2 = heavy).

Figure 1 shows health behavior trajectories. Four trajectories were identified for physical activity: persistent inactivity (15.4%), intermediate then inactivity (35.7%), intermediate then recommended (22.5%), persistently recommended level (26.5%). Five trajectories of fruit and vegetable consumption were identified: persistent low (13.7%), low then intermediate (12.3%), persistent intermediate (48.0%), intermediate then high (15.7%), persistent high (10.3%). There were four trajectories for smoking: never (47.5%), long-term ex-smoker (quit smoking before 1985–1988, 34.2%), recent ex-smoker (quit smoking during the follow-up, 6.5%), persistent smoker (11.8%). Five trajectories of alcohol consumption were identified: never (10.5%), moderate then none (7.8%), persistent moderate (63.0%), moderate then heavy (9.4%), persistent heavy (9.3%). Mean posterior probabilities were comprised between 0.73–0.74 for physical activity, 0.74–0.85 for fruit and vegetable, 0.90–0.99 for smoking, and 0.86–0.95 for alcohol.

Participants with at least one assessment of health behaviors and disability were eligible (*N* = 7,431). Participants not assigned to at least one trajectory (*n* = 7), with missing cognitive (*n* = 485) or disability data (*n* = 188) were excluded; analyses are based on 6,825 participants. Compared to them, those excluded (*n* = 606, 8.2%) were older (64.9 years vs 63.9 years, sex-adjusted *p* < .001), and more likely to be women (42.2% vs 29.1%, age-adjusted *p* < .001) or from the lower socioeconomic group (27.4% vs 10.9%, age/sex-adjusted *p* < .001). However, they had a similar frequency of disability (22.7% vs 19.2%, age/sex-adjusted *p* = 0.33).

Supplementary Table 1 shows participants’ characteristics; 1,310 participants (19.2%) were disabled at least once. They were older, less likely to be men, married or cohabiting, and from the lower socioeconomic group. They were also less active, consumed fewer fruit and vegetable, were more often persistent/recent ex-smokers, alcohol abstainers, or persistent heavy drinkers, and had a poorer health profile.

Analysis on health behaviors considered in separate models (Table 1, Model 1) showed that participants with persistent physical inactivity or intermediate then inactivity (unhealthy trajectories) had a higher disability risk than those with persistent recommended activity. Only participants with persistent low fruit and vegetable consumption (unhealthy trajectory) had a higher disability risk compared with participants with persistent high consumption. Persistent smokers and recent ex-smokers (unhealthy trajectories) had an increased disability risk compared with never smokers. Never, moderate then none, or persistent heavy drinkers (unhealthy trajectories) had a higher disability risk compared with persistent moderate drinkers. Interactions between sex or age and health behaviors were not statistically significant. Results for Model 2 (all health behaviors included) and Model 3 (additionally adjusted for covariates) were similar for physical activity and smoking, whereas the association with fruit and vegetable consumption disappeared and that with alcohol was slightly attenuated. Analyses of healthy/unhealthy trajectories show that physical inactivity had the strongest association and fruit and vegetable the weakest.

**Table 1.** Health Behavior Trajectories (1985–2004) and Subsequent Disability (2006–2013)

Trajectory	*n* Disabled/*N*	Model 1^a^				Model 2^b^				Model 3^c^			
		OR	95% CI	*p*	*p* ^d^	OR	95% CI	*p*	*p* ^d^	OR	95% CI	*p*	*p* ^d^
**Physical activity**													
Persistent inactivity	253/804	2.38	1.92–2.94	<.001		2.23	1.80–2.77	<.001		1.78	1.41–2.24	<.001	
Intermediate then inactivity	513/2,370	1.77	1.50–2.09	<.001		1.72	1.46–2.03	<.001		1.58	1.32–1.88	<.001	
Intermediate then recommended	263/1,519	1.12	0.93–1.36	.24		1.12	0.93–1.36	.24		1.08	0.89–1.32	.42	
Persistent recommended	281/2,132	1.00	(Ref.)		<.001	1.00	(Ref.)		<.001	1.00	(Ref.)		<0.001
*Persistent inactivity, intermediate then inactivity vs intermediate then recommended, persistent recommended*		1.82	1.60–2.07	<.001		1.76	1.54–2.00	<.001		1.58	1.38–1.82	<.001	
**Consumption of fruit and vegetable**													
Persistent low	197/905	1.47	1.15–1.89	.003		1.15	0.89–1.49	.28		1.16	0.89–1.52	.27	
Low then intermediate	150/759	1.23	0.94–1.59	.13		1.03	0.79–1.35	.80		1.13	0.86–1.49	.37	
Persistent intermediate	617/3,157	1.12	0.91–1.38	.27		1.02	0.83–1.26	.82		1.06	0.86–1.32	.57	
Intermediate then high	200/1,217	0.96	0.76–1.23	.76		0.94	0.74–1.20	.63		1.05	0.82–1.35	.70	
Persistent high	146/787	1.00	(Ref.)		.006	1.00	(Ref.)		.59	1.00	(Ref.)		.82
*Persistent low vs all other*		1.35	1.13–1.61	.001		1.17	0.98–1.41	.09		1.11	0.92–1.35	.27	
**Smoking**													
Never	606/3,367	1.00	(Ref.)			1.00	(Ref.)			1.00	(Ref.)		
Long-term ex-smoker	443/2,498	1.03	0.89–1.18	.73		1.06	0.91–1.22	.47		0.94	0.81–1.10	.45	
Recent ex-smoker	94/390	1.46	1.13–1.88	.004		1.46	1.13–1.90	.004		1.25	0.94–1.66	.13	
Persistent	167/570	1.93	1.57–2.37	<.001	<.001	1.80	1.46–2.23	<.001	<.001	1.73	1.39–2.16	<.001	<.001
*Persistent or recent ex-smoker vs never or long-term ex-smoker*		1.71	1.45–2.01	<.001		1.63	1.38–1.92	<.001		1.57	1.32–1.87	<.001	
**Alcohol**													
Never	139/531	1.29	1.04–1.60	.02		1.25	1.00–1.55	.05		1.06	0.84–1.35	.60	
Moderate then none	148/464	1.61	1.30–2.01	<.001		1.53	1.23–1.90	<.001		1.39	1.09–1.76	.007	
Persistent moderate	778/4,517	1.00	(Ref.)			1.00	(Ref.)			1.00	(Ref.)		
Moderate then heavy	115/678	1.04	0.83–1.30	.76		1.03	0.82–1.29	.79		1.02	0.82–1.28	.85	
Persistent heavy	130/635	1.36	1.10–1.68	.004	<.001	1.24	1.00–1.54	.05	.002	1.13	0.90–1.43	.29	.13
*Never, moderate then none, persistent heavy vs persistent moderate, moderate then heavy*		1.41	1.23–1.61	<.001		1.33	1.16–1.53	<.001		1.19	1.02–1.38	.02	

*Note:* CI = confidence interval; OR = odds ratio.

^a^Adjusted for sex, age, marital status, socioeconomic status, time, time × age.

^b^Model 1 + other health behavior trajectories.

^c^Model 2 + time-dependent body mass index, mini-mental state examination, depressive symptoms, antidepressant drugs, diabetes, anti-inflammatory drugs for joint pain, cancer, osteoarthritis, Parkinson’s disease, stroke, coronary heart disease, hypertension, hypercholesterolemia.

^d^Global test.

**Figure 2.**
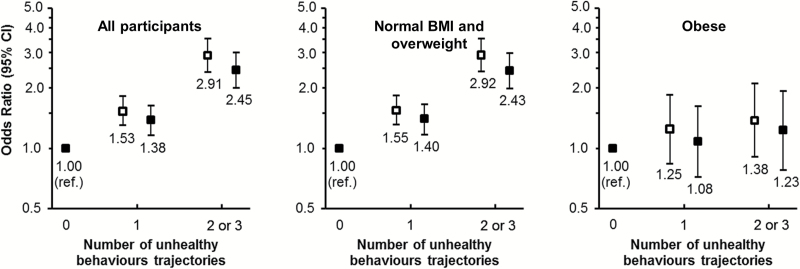
Odds ratios (95% confidence intervals) of disability (2006–2013) according to the unhealthy behavior trajectories score (1985–2004). Adjusted for sex, age, marital status, socioeconomic status, time, time×age (empty), and additionally adjusted for time-dependent body mass index, mini-mental state examination, depressive symptoms, antidepressant drugs, diabetes, anti-inflammatory drugs for joint pain, cancer, osteoarthritis, Parkinson’s disease, stroke, coronary heart disease, hypertension, hypercholesterolemia (full).

According to the unhealthy behavior trajectories score, 38% of participants had no unhealthy trajectory, 42% had one, and 20% had two or three. Unhealthy trajectories were less frequent in older participants and men; higher education, being married or cohabiting, and a better health profile were associated with fewer unhealthy trajectories (Supplementary Table 2). Disability risk increased with the score (Figure 2); in all participants, the odds ratio (OR) per unit increase was 1.64 (95% confidence interval [CI] = 1.50–1.79, *p* < .001; OR = 1.47, 95% CI = 1.34–1.62, *p* < .001, after adjustment for time-dependent covariates). The relation between the score and disability was significantly modified by obesity (*p*-interaction = .002); in nonobese persons, the OR per unit increase was 1.71 (95% CI = 1.55–1.89; *p* < .001), whereas in obese persons it was 1.17 (95% CI = 0.95–1.44; *p* = .13). In Model 3, all unhealthy behaviors, except fruit and vegetable consumption, were associated with disability in nonobese participants, whereas in obese participants none of them was associated.

All unhealthy trajectories were associated with mobility disability; there was no association with fruit and vegetable with ADL/IADL disability (Supplementary Table 3).

**Figure 3.**
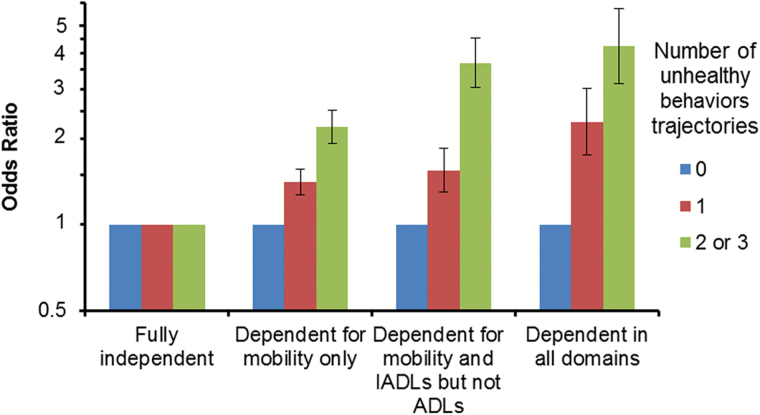
Association of the unhealthy behavior trajectories score (1985–2004) with the hierarchical disability indicator (2006–2013). Odds ratios (95% confidence intervals) computed using multinomial GEE models adjusted for sex, age, marital status, socioeconomic status, time, time × age.

Analyses based on multinomial GEE (Figure 3) showed that for a given number of unhealthy behaviors, the strength of the association increased with increasing disability, and that for each level of disability, the strength of the association increased with the number of unhealthy trajectories.

Results using inversely probability-weighted GEE were similar to our main findings (Supplementary Table 4). Excluding participants with a single health behavior assessment (1%) did not change our conclusions.

## Discussion

Unhealthy trajectories of physical activity, smoking, and alcohol consumption over midlife to early old age were independently associated with increased subsequent disability risk. Disability risk increased progressively with the number of unhealthy trajectories. There is already evidence showing unhealthy diet (22), smoking (23,24), alcohol abstinence (25), physical inactivity (26,27), or decreasing physical activity (28,29) in midlife to increase disability risk at older ages. However, these studies did not take into account multiple behaviors simultaneously, potentially overestimating the effect of single health behaviors as unhealthy behaviors tend to cluster (30,31). Fewer studies have considered multiple midlife behaviors, showing them to be associated with locomotor disability in men (32) and ADL disability (9,33).

To the best of our knowledge, no study has examined the association of multiple health behavior trajectories in relation to disability. One previous study examined changes in health behaviors and disability transitions simultaneously and found healthy behaviors to be associated with remaining functionally independent (34). However, as health behaviors and disability were assessed simultaneously, reverse causation may have led participants to change health behaviors due to the development of disability. In our study, health behaviors were assessed starting in midlife, before the first signs of disability, making it less prone to bias from reverse causation. Moreover, the period between midlife and early old age involves many work and family related transitions, such as transition from work to retirement, which may modify lifestyle (35). Studies based on a single assessment of health behaviors in old age may therefore suffer from exposure misclassification due to lifestyle changes induced by disease or other life-related events.

In this study, four trajectories of physical activity were identified and participants with persistent inactivity or intermediate physical activity who reduced their activity over the follow-up were at higher disability risk compared to participants with persistent recommended level. Participants with intermediate physical activity who increased their activity over the follow-up had a similar disability risk. Therefore, increasing one’s physical activity in midlife or having persistently recommended activity may reduce disability risk in old age.

Four trajectories were identified for smoking; long-term ex-/never smokers had lower disability risk. Risk in recent ex-smokers was lower than in current smokers but remained elevated compared with long-term ex-/never-smokers. We identified five trajectories for alcohol; compared to participants with persistent moderate consumption, those with moderate consumption who stopped drinking were at highest disability risk. It is possible that alcohol cessation is due to the occurrence of diseases causing disability. Alcohol abstainers over 20 years of follow-up also had higher disability risk. Alcohol abstinence has been previously associated with disability (36,37), which may partly be explained by inclusion of heavy drinkers who stopped drinking (38). Persistent heavy drinkers had a higher disability risk. Fruit and vegetable consumption was not associated with disability after adjusting for other health behaviors, in particular physical activity; the exceptions being associations with mobility disability. Adjustment for covariates attenuated the associations of unhealthy behavior trajectories with disability, though not completely, with depressive symptoms, cardiovascular disease and their risks factors, and BMI playing an important role. Unhealthy behavior trajectories were associated with disability in nonobese participants but not in obese; therefore, obesity appears to blunt the association of health behaviors with disability.

Our findings need to be considered in light of some limitations. First, questionnaires used to assess health behaviors were imperfect. Diet is particularly difficult to measure, especially over a long follow-up; this could perhaps explain the absence of association with our dietary behavior indicator. Second, a small proportion of participants were excluded from the analyses (8%) but had a similar disability risk compared with those included; this is therefore unlikely to have affected our findings. Third, we may have underestimated the role of some comorbidities, in particular stroke, because participants with severe stroke during the follow-up may have dropped out from the study. However, our conclusions remained unchanged in sensitivity analyses that took into account death and dropout.

This study’s main strengths include its large size and length of follow-up with regular health behavior assessments, allowing the definition of distinct trajectories. The main outcome is a hierarchical indicator of disability that combines information from three disability scales ordered in a hierarchy and describes the progression of disability (12).

Our findings have important public health implications, as behaviors are potentially modifiable and interventions aimed at promoting a healthy lifestyle in midlife may help to prevent subsequent disability. These findings may be useful for policy-makers informing the potential benefit of multibehavior interventions (39) in midlife compared to interventions at older ages (40,41).

## Supplementary Material

Please visit the article online at http://gerontologist.oxfordjournals.org/ to view supplementary material.

## Funding

Work supported by US National Institutes of Health (R01AG013196; R01AG034454, R01HL036310); UK Medical Research Council (K013351); and Economic and Social Research Council (ES/ J023299).

## Conflict of Interest

None.

## Supplementary Material

Supplementary Data
