# Supplementary material for: Trajectories of Unhealthy Behaviors in Midlife and Risk of Disability at Older Ages in the Whitehall II Cohort Study
Source: J Gerontol A Biol Sci Med Sci. 2016 Mar 30;71(11):1500–6. doi: 10.1093/gerona/glw060 (PMC5055647; doi:10.1093/gerona/glw060)
Supplement: Supplementary Data [file supp_71_11_1500__index.html]

Trajectories of Unhealthy Behaviors in Midlife and Risk of Disability at Older Ages in the Whitehall II Cohort Study — Trajectories of Unhealthy Behaviors in Midlife and Risk of Disability at Older Ages in the Whitehall II Cohort Study — Supplementary Data 

# Trajectories of Unhealthy Behaviors in Midlife and Risk of Disability at Older Ages in the Whitehall II Cohort Study

## Supplementary Data

Data files

- Supplementary Data - Supplementary Data
