# Supplementary material for: Trajectories of Unhealthy Behaviors in Midlife and Risk of Disability at Older Ages in the Whitehall II Cohort Study
Source: J Gerontol A Biol Sci Med Sci. 2016 Mar 30;71(11):1500–6. doi: 10.1093/gerona/glw060 (PMC5055647; doi:10.1093/gerona/glw060)
Supplement: Supplementary Data [file supp_glw060_Supplementary_rev.docx]

**Supplementary Table 1. Participants’ Characteristics**

|  | **Overall** | **Not disabled** | **Disabled at least once** | **P^a^ (age- and sex- adjusted)** |
| --- | --- | --- | --- | --- |
| N (%) | 6825 | 5515 (80.8) | 1310 (19.2) |  |
| Age in 2006 (years), mean (SD) | 63.9 (6.0) | 63.4 (5.8) | 66.1 (6.1) | <0.001 |
| Men | 4837 (70.9) | 4068 (73.8) | 769 (58.7) | <0.001 |
| Married, cohabiting | 5114 (74.9) | 4261 (77.3) | 853 (65.1) | <0.001 |
| High Socioeconomic status | 3117 (45.7) | 2645 (48.0) | 472 (36.0) | <0.001 |
| **Trajectories of health behaviors (1985-88 to 2002-04)** | | | | |
| Physical activity | |  |  |  |
| Persistent inactivity | 804 (11.8) | 551 (10.0) | 253 (19.3) |  |
| Intermediate then inactivity | 2370 (34.7) | 1857 (33.7) | 513 (39.2) |  |
| Intermediate then recommended | 1519 (22.3) | 1256 (22.8) | 263 (20.1) |  |
| Persistent recommended | 2132 (31.2) | 1851 (33.6) | 281 (21.5) | <0.001 |
| Consumption of fruit and vegetable | | | |  |
| Persistent low | 905 (13.3) | 708 (12.8) | 197 (15.0) |  |
| Low then intermediate | 759 (11.1) | 609 (11.0) | 150 (11.5) |  |
| Persistent intermediate | 3157 (46.3) | 2540 (46.1) | 617 (47.1) |  |
| Intermediate then high | 1217 (17.8) | 1017 (18.4) | 200 (15.3) |  |
| Persistent high | 787 (11.5) | 641 (11.6) | 146 (11.1) | <0.001 |
| Smoking | |  |  |  |
| Never | 3367 (49.3) | 2761 (50.1) | 606 (46.3) |  |
| Long-term ex-smoker | 2498 (36.6) | 2055 (37.3) | 443 (33.8) |  |
| Recent ex-smoker | 390 (5.7) | 296 (5.4) | 94 (7.2) |  |
| Persistent | 570 (8.4) | 403 (7.3) | 167 (12.7) | <0.001 |
| Alcohol |  |  |  |  |
| Never | 531 (7.8) | 392 (7.1) | 139 (10.6) |  |
| Moderate then none | 464 (6.8) | 316 (5.7) | 148 (11.3) |  |
| Persistent moderate | 4517 (66.2) | 3739 (67.8) | 778 (59.4) |  |
| Moderate then heavy | 678 (9.9) | 563 (10.2) | 115 (8.8) |  |
| Persistent heavy | 635 (9.3) | 505 (9.2) | 130 (9.9) | <0.001 |

**Supplementary Table 1 Follows**

|  | **Overall** | **Not disabled** | **Disabled at least once** | **P^a^ (age- and sex- adjusted)** |
| --- | --- | --- | --- | --- |
| **Time-dependent covariates** |  |  |  |  |
| BMI (kg/m²), mean (SD)^b^ | 26.0 (3.8) | 25.6 (3.5) | 27.4 (4.6) | <0.001 |
| MMSE score, mean (SD)^b^ | 28.4 (1.2) | 28.4 (1.2) | 28.0 (1.5) | <0.001 |
| Depressive symptoms^c^ | 2243 (32.9) | 1634 (29.6) | 609 (46.5) | <0.001 |
| Use of antidepressant drugs^c^ | 592 (8.7) | 372 (6.7) | 220 (16.8) | <0.001 |
| Bone fracture^c^ | 1472 (21.6) | 1126 (20.4) | 346 (26.4) | 0.001 |
| Diabetes^c^ | 929 (13.6) | 650 (11.8) | 279 (21.3) | <0.001 |
| Use of anti-inflammatory drugs^c^ | 1093 (16.0) | 728 (13.2) | 365 (27.9) | <0.001 |
| Cancer^c^ | 941 (13.8) | 717 (13.0) | 224 (17.1) | 0.06 |
| Osteoarthritis^c^ | 2787 (40.8) | 1975 (35.8) | 812 (62.0) | <0.001 |
| Parkinson’s disease^c^ | 50 (0.7) | 10 (0.2) | 40 (3.1) | <0.001 |
| Stroke^c^ | 234 (3.4) | 157 (2.8) | 77 (5.9) | <0.001 |
| CHD^c^ | 1587 (23.3) | 1085 (19.7) | 502 (38.3) | <0.001 |
| Hypertension^c^ | 4252 (62.3) | 3292 (59.7) | 960 (73.3) | <0.001 |
| Hypercholesterolemia^c^ | 3689 (54.1) | 2900 (52.6) | 789 (60.2) | 0.006 |

BMI: Body Mass Index; MMSE: Mini-Mental State Examination; SD: Standard Deviation; CHD: Coronary Heart Disease.

Values are numbers (%) unless stated otherwise.

^a^ Analysis of covariance for continuous variables and Mantel-Haenszel chi-square test for categorical variables.

^b^ Mean of all measures taken between 1985-88 and 2012-13.

^c^ At least one report between 1985-88 and 2012-13.

**Supplementary Table 2. Characteristics of the participants according to number of unhealthy** **behaviors trajectories**

|  | **Number of unhealthy trajectories** | | | **P^a^ (age- and sex- adjusted)** |
| --- | --- | --- | --- | --- |
|  | **0** | **1** | **2 or 3** |  |
| N (%) | 2597 (38.1) | 2873 (42.1) | 1355 (19.9) |  |
| Age in 2006 (years), mean (SD) | 64.2 (6.0) | 63.6 (5.9) | 63.7 (6.0) | <0.001 |
| Male sex | 2109 (81.2) | 1980 (68.9) | 748 (55.2) | <0.001 |
| Marital status |  |  |  |  |
| Married, cohabiting | 2178 (83.9) | 2100 (73.1) | 836 (61.7) |  |
| Divorced, separated, widowed | 246 (9.5) | 374 (13.0) | 264 (19.5) |  |
| Single | 173 (6.7) | 399 (13.9) | 255 (18.8) | <0.001 |
| Socioeconomic status |  |  |  |  |
| High (administrative) | 1463 (56.3) | 1282 (44.6) | 372 (27.5) |  |
| Intermediate (professional or executive) | 1015 (39.1) | 1283 (44.7) | 667 (49.2) |  |
| Low (clerical or support) | 119 (4.6) | 308 (10.7) | 316 (23.3) | <0.001 |
| **Trajectories of health behaviors (1985-88 to 2002-04)** | | |  |  |
| Physical activity | |  |  |  |
| Persistent inactivity | 0 (0) | 421 (14.7) | 383 (28.3) |  |
| Intermediate then inactivity | 0 (0) | 1518 (52.8) | 852 (62.9) |  |
| Intermediate then recommended | 1078 (41.5) | 386 (13.4) | 55 (4.1) |  |
| Persistent recommended | 1519 (58.5) | 548 (19.1) | 65 (4.8) | <0.001 |
| Consumption of fruit and vegetable | | | |  |
| Persistent low | 226 (8.7) | 391 (13.6) | 288 (21.3) |  |
| Low then intermediate | 252 (9.7) | 314 (10.9) | 193 (14.2) |  |
| Persistent intermediate | 1169 (45.0) | 1378 (48.0) | 610 (45.0) |  |
| Intermediate then high | 574 (22.1) | 483 (16.8) | 160 (11.8) |  |
| Persistent high | 376 (14.5) | 307 (10.7) | 104 (7.7) | <0.001 |
| Smoking | |  |  |  |
| Never | 1407 (54.2) | 1486 (51.7) | 474 (35.0) |  |
| Long-term ex-smoker | 1190 (45.8) | 1065 (37.1) | 243 (17.9) |  |
| Recent ex-smoker | 0 (0) | 156 (5.4) | 234 (17.3) |  |
| Persistent | 0 (0) | 166 (5.8) | 404 (29.8) | <0.001 |
| Alcohol |  |  |  |  |
| Never | 0 (0) | 179 (6.2) | 352 (26.0) |  |
| Moderate then none | 0 (0) | 151 (5.3) | 313 (23.1) |  |
| Persistent drinker | 2259 (87.0) | 1966 (68.4) | 292 (21.5) |  |
| Moderate then heavy | 338 (13.0) | 295 (10.3) | 45 (3.3) |  |
| Persistent heavy | 0 (0) | 282 (9.8) | 353 (26.1) | <0.001 |

**Supplementary Table 2 Follows**

|  | **Number of unhealthy behaviors trajectories** | | | **P^a^ (age- and sex- adjusted)** |
| --- | --- | --- | --- | --- |
|  | **0** | **1** | **2 or 3** |  |
| **Time-dependent covariates** |  |  |  |  |
| BMI (m/kg²), mean (SD)^b^ | 25.6 (3.4) | 26.0 (3.8) | 26.7 (4.4) | <0.001 |
| MMSE score, mean (SD)^b^ | 28.5 (1.1) | 28.4 (1.3) | 28.1 (1.4) | <0.001 |
| Depressive symptoms^c^ | 696 (26.8) | 987 (34.4) | 560 (41.3) | <0.001 |
| Use of antidepressant drugs^c^ | 156 (6.0) | 277 (9.6) | 159 (11.7) | <0.001 |
| Bone fracture^c^ | 540 (20.8) | 635 (22.1) | 297 (21.9) | 0.17 |
| Diabetes^c^ | 259 (10.0) | 385 (13.4) | 285 (21.0) | <0.001 |
| Use of anti-inflammatory drugs^c^ | 388 (14.9) | 453 (15.8) | 252 (18.6) | 0.12 |
| Cancer^c^ | 420 (16.2) | 381 (13.3) | 140 (10.3) | <0.001 |
| Osteoarthritis^c^ | 1025 (39.5) | 1166 (40.6) | 596 (44.0) | 0.94 |
| Parkinson’s disease^c^ | 13 (0.5) | 27 (0.9) | 10 (0.7) | 0.06 |
| Stroke^c^ | 82 (3.2) | 98 (3.4) | 54 (4.0) | 0.01 |
| CHD^c^ | 532 (20.5) | 634 (22.1) | 421 (31.1) | <0.001 |
| Hypertension^c^ | 1567 (60.3) | 1773 (61.7) | 912 (67.3) | <0.001 |
| Hypercholesterolemia^c^ | 1337 (51.5) | 1554 (54.1) | 798 (58.9) | <0.001 |

Values are numbers (%) unless stated otherwise.

^a^ Analysis of covariance for continuous variables and Mantel-Haenszel chi-square test for categorical variables.

^b^ Mean of all measures taken between 1985-88 and 2012-13.

^c^ At least one report between 1985-88 and 2012-13.

**Supplementary Table 3. Associations of trajectories of health behaviors with mobility disability and disability in basic or instrumental activities of daily living.**

|  | **Model 1^a^** | |  | **Model 2^b^** | |  | **Model 3^c^** | |
| --- | --- | --- | --- | --- | --- | --- | --- | --- |
| **Trajectory** | **OR (95% CI)** | **P** |  | **OR (95% CI)** | **P** |  | **OR (95% CI)** | **P** |
| **Mobility^d^** |  |  |  |  |  |  |  |  |
| **Physical activity** |  |  |  |  |  |  |  |  |
| Unhealthy *vs.* healthy^e^ | 1.59 (1.46, 1.74) | <0.001 |  | 1.52 (1.39, 1.66) | <0.001 |  | 1.40 (1.28, 1.54) | <0.001 |
| **Consumption of fruit and vegetable** | |  |  |  |  |  |  |  |
| Unhealthy *vs.* healthy^e^ | 1.73 (1.51, 1.97) | <0.001 |  | 1.54 (1.34, 1.76) | <0.001 |  | 1.52 (1.33, 1.74) | <0.001 |
| **Smoking** |  |  |  |  |  |  |  |  |
| Unhealthy *vs.* healthy^e^ | 1.97 (1.74, 2.24) | <0.001 |  | 1.85 (1.63, 2.10) | <0.001 |  | 1.76 (1.55, 2.01) | <0.001 |
| **Alcohol** |  |  |  |  |  |  |  |  |
| Unhealthy *vs.* healthy^e^ | 1.36 (1.22, 1.50) | <0.001 |  | 1.28 (1.16, 1.42) | <0.001 |  | 1.16 (1.04, 1.29) | 0.006 |
| **Number of unhealthy behaviors trajectories^f^** | | |  |  |  |  |  |  |
| 0 | 1.00 (Ref.) | -- |  | -- | -- |  | 1.00 (Ref.) | -- |
| 1 | 1.45 (1.32, 1.60) | <0.001 |  | -- | -- |  | 1.37 (1.24, 1.51) | <0.001 |
| 2 or 3 | 2.49 (2.19, 2.82) | <0.001 |  | -- | -- |  | 2.08 (1.83, 2.37) | <0.001 |
| Trend (per one unhealthy trajectory) | 1.56 (1.47, 1.66) | <0.001 |  | -- | -- |  | 1.43 (1.34, 1.53) | <0.001 |
| **Number of unhealthy behaviors trajectories^g^** | | |  |  |  |  |  |  |
| 0 | 1.00 (Ref.) | -- |  |  |  |  | 1.00 (Ref.) | -- |
| 1 | 1.51 (1.36, 1.68) | <0.001 |  | -- | -- |  | 1.43 (1.29, 1.59) | <0.001 |
| 2 | 2.25 (1.98, 2.55) | <0.001 |  | -- | -- |  | 1.94 (1.70, 2.20) | <0.001 |
| 3 or 4 | 3.67 (3.00, 4.48) | <0.001 |  | -- | -- |  | 3.06 (2.49, 3.77) | <0.001 |
| Trend (per one unhealthy trajectory) | 1.52 (1.44, 1.60) | <0.001 |  | -- | -- |  | 1.42 (1.35, 1.50) | <0.001 |
|  |  |  |  |  |  |  |  |  |
| **ADL/IADL^h^** |  |  |  |  |  |  |  |  |
| **Physical activity** |  |  |  |  |  |  |  |  |
| Unhealthy *vs.* healthy^e^ | 1.58 (1.41, 1.76) | <0.001 |  | 1.53 (1.37, 1.70) | <0.001 |  | 1.37 (1.22, 1.54) | <0.001 |
| **Consumption of fruit and vegetable** | |  |  |  |  |  |  |  |
| Unhealthy *vs.* healthy^e^ | 1.30 (1.11, 1.51) | 0.001 |  | 1.16 (0.99, 1.35) | 0.07 |  | 1.10 (0.93, 1.29) | 0.26 |
| **Smoking** |  |  |  |  |  |  |  |  |
| Unhealthy *vs.* healthy^e^ | 1.62 (1.41, 1.87) | <0.001 |  | 1.56 (1.35, 1.79) | <0.001 |  | 1.50 (1.29, 1.74) | <0.001 |
| **Alcohol** |  |  |  |  |  |  |  |  |
| Unhealthy *vs.* healthy^e^ | 1.34 (1.19, 1.52) | <0.001 |  | 1.29 (1.14, 1.45) | <0.001 |  | 1.18 (1.04, 1.34) | 0.01 |
| **Number of unhealthy behaviors trajectories^f^** | | |  |  |  |  |  |  |
| 0 | 1.00 (Ref.) | -- |  | -- | -- |  | 1.00 (Ref.) | -- |
| 1 | 1.34 (1.18, 1.52) | <0.001 |  | -- | -- |  | 1.21 (1.06, 1.38) | 0.004 |
| 2 or 3 | 2.26 (1.96, 2.62) | <0.001 |  | -- | -- |  | 1.88 (1.61, 2.19) | <0.001 |
| Trend (per one unhealthy trajectory) | 1.50 (1.39, 1.62) | <0.001 |  | -- | -- |  | 1.36 (1.26, 1.48) | <0.001 |

^a^ Adjusted for sex, age in 2006, marital status, socioeconomic status, time, time×age.

^b^ Adjusted for sex, age in 2006, marital status, socioeconomic status, time, time×age, and other behaviors trajectories.

^c^ Adjusted for sex, age in 2006, marital status, socioeconomic status, time, time×age, trajectories of other health behaviors, and time-dependent covariates (BMI, MMSE score, depressive symptoms, use of antidepressant drugs, diabetes, use of anti-inflammatory drugs for joint pain, cancer, osteoarthritis, Parkinson’s disease, stroke, coronary heart disease, hypertension, hypercholesterolemia).

^d^ Of 6,871 participants included in these analyses, 3,825 (55.7%) developed mobility disability at least once during the follow-up.

^e^ Definitions of unhealthy and healthy trajectories are reported in Table 1.

^f^ The number of unhealthy behaviors trajectories was computed without consumption of fruit and vegetable for comparability with main results.

^g^ The number of unhealthy behaviors trajectories was computed with consumption of fruit and vegetable.

^h^ Of 6,872 participants included in these analyses, 1,828 (26.6%) developed disability in ADL/IADL at least once during the follow-up.

**Supplementary Table 4. Association of Trajectories of Health Behaviors (1985-2004) With Subsequent Disability (2006-2013) using inverse-probability weighted GEE (N=6142)**

|  | **Non-weighted GEE** | |  | **Weighted GEE** | |
| --- | --- | --- | --- | --- | --- |
| **Trajectory** | **OR (95% CI)** | **P** |  | **OR (95% CI)** | **P** |
| **Physical activity^a^** |  |  |  |  |  |
| Unhealthy *vs.* healthy^b^ | 1.78 (1.55, 2.05) | <0.001 |  | 1.77 (1.53, 2.05) | <0.001 |
| **Consumption of fruit and vegetable^a^** |  |  |  |  |  |
| Unhealthy *vs.* healthy^b^ | 1.20 (0.99, 1.46) | 0.07 |  | 1.16 (0.94, 1.42) | 0.16 |
| **Smoking^a^** |  |  |  |  |  |
| Unhealthy *vs.* healthy^b^ | 1.65 (1.38, 1.97) | <0.001 |  | 1.63 (1.35, 1.97) | <0.001 |
| **Alcohol^a^** |  |  |  |  |  |
| Unhealthy *vs.* healthy^b^ | 1.33 (1.15, 1.55) | <0.001 |  | 1.28 (1.10, 1.50) | 0.002 |
| **Number of unhealthy behaviors trajectories^c^** | | | | | |
| 0 | 1.00 (Ref.) | -- |  | 1.00 (Ref.) | -- |
| 1 | 1.55 (1.32, 1.83) | <0.001 |  | 1.54 (1.30, 1.83) | <0.001 |
| 2-3 | 2.72 (2.26, 3.27) | <0.001 |  | 2.59 (2.14, 3.14) | <0.001 |
| Trend (per one unhealthy trajectory) | 1.65 (1.50, 1.81) | <0.001 |  | 1.61 (1.46, 1.78) | <0.001 |

^a^ Adjusted for continuous age in 2006, marital status, socioeconomic status, time, and other behaviors trajectories.

^b^ Definitions of unhealthy and healthy trajectories as in Table 1.

^c^ Adjusted for continuous age in 2006, marital status, socioeconomic status, and time.
